# A Rare Case of Epithelioid Hemangioma Presenting as an Isolated Sacral Mass

**DOI:** 10.7759/cureus.29801

**Published:** 2022-10-01

**Authors:** Akriti Pokhrel, Kiron V Nair, Shaun U Din, Akhtar Cheema, Vladimir Gotlieb

**Affiliations:** 1 Internal Medicine, Brookdale University Hospital Medical Center, Brooklyn, USA; 2 Hematology and Medical Oncology, Brookdale University Hospital Medical Center, Brooklyn, USA; 3 Radiation Oncology, Brookdale University Hospital Medical Center, Brooklyn, USA; 4 Pathology, Brookdale University Hospital Medical Center, Brooklyn, USA; 5 Department of Hematology/Oncology, Brookdale University Hospital Medical Center, Brooklyn, USA

**Keywords:** epithelioid hemangioma, primary intraosseous, vascular malformation, sacrum, perivascular epithelioid cell neoplasms, nerve compression syndromes

## Abstract

Epithelioid hemangioma (EH) is an uncommon benign vascular tumor of mesenchymal origin. It mainly presents as expanding nodules around the ear, the forehead, and long bones. Only a handful of cases have been found in cervical, thoracic, lumbar, and sacral vertebrae as lytic lesions with pain and neurological impairment. We present the case of a 36-year-old female with an incidental finding of a sacral mass along with inguinal lymphadenopathy on imaging. Initially, there were no symptoms. The mass gradually progressed and later showed an extraosseous extension with involvement of sacral neural foramina and nerve roots causing severe low back pain and weakness of the left lower extremity. Differential diagnoses initially included secondary metastases and chordoma. However, the biopsy of the mass revealed findings consistent with an EH. To our knowledge, this is the first case of EH presenting as an isolated mass in the sacrum and the third case of EH involving the sacrum in continuation with other vertebrae. EH should be in our differential diagnoses when there is a sacral mass.

## Introduction

Epithelioid hemangioma (EH) is a benign vascular tumor. It commonly affects people aged 20-50 years, with no gender predilection overall [1]; however, a male predominance (1.4:1) has been reported in the osseous form of the disease [2]. Generally, it presents as an expanding smooth-surfaced nodule. It is usually located in the head and neck area, especially in the region around the ear (approximately 85% of cases) and forehead, but less commonly can also involve the long bones of the arm and legs, oral cavity, penis, and lymph nodes [3,4]. Bones involved are long tubular bones (around 40%), short tubular bones (more commonly feet), flat bones, and, rarely, vertebrae [3]. We could only find a handful of related case reports [2,5,6]. In the spine, EH can cause pain, instability, and/or neurologic dysfunction. On radiology (plain and computed tomography (CT) scan), it presents as a lytic, well-defined lesion. Magnetic resonance imaging (MRI) shows typically hypointense on T1-weighted axial imaging (T1WI), hyperintense on T2-weighted axial imaging (T2WI), and avidly enhancing, often with an extraosseous soft-tissue component [2]. Although the World Health Organization (WHO) classifies EH as a benign tumor, it can display locally aggressive features. The WHO classification of vascular tumors of the bone consists of benign (hemangioma), intermediate, locally aggressive/rarely metastatic as EH, or malignant (angiosarcoma and epithelioid hemangioendothelioma (EHE)) [7]. Because of its unusual cytologic appearance and growth patterns, EH is often confused with a malignant tumor [8].

Here, we report a rare case of a 36-year-old female who was incidentally found to have a sacral mass on imaging which was later consistent with an EH on biopsy. To our knowledge, this is the first case of sacral EH without the involvement of other vertebrae and the third case of vertebral EH in continuation with other vertebrae.

## Case presentation

A 36-year-old female with a medical history of diabetes, asthma, and hypertension presented with generalized swelling of the body and shortness of breath for three days.

Vital signs were stable except for blood pressure of 220/110 mmHg. Physical examination was significant for pitting edema of bilateral lower extremities. Laboratory tests were unremarkable. Chest X-ray was suggestive of pulmonary vascular congestion. The patient was admitted for a hypertensive emergency.

**Figure 1:**
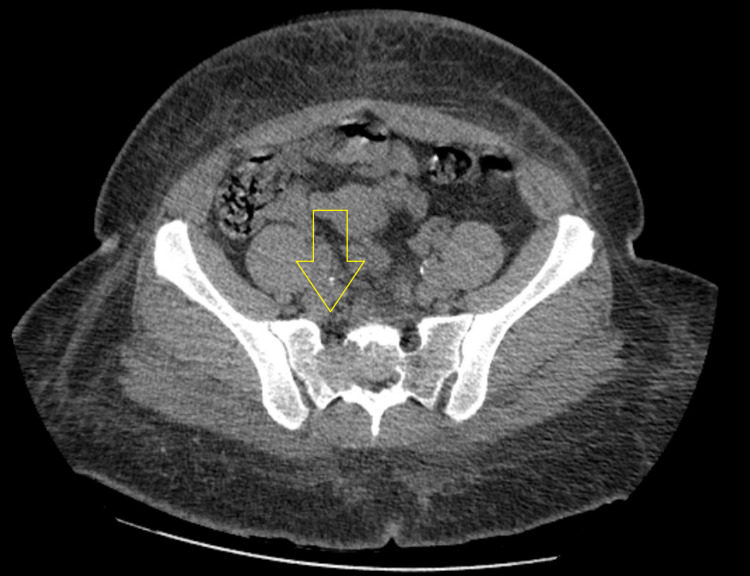
Computed tomography of the pelvis showing a lytic mass within the right sacrum with involved sacral neural foramen and anterior cortical erosion.

In the inpatient unit, the patient had diffuse abdominal pain. To determine the cause, a CT scan of the abdomen and pelvis without contrast was done which showed a sacral mass (Figure 1).

**Figure 2:**
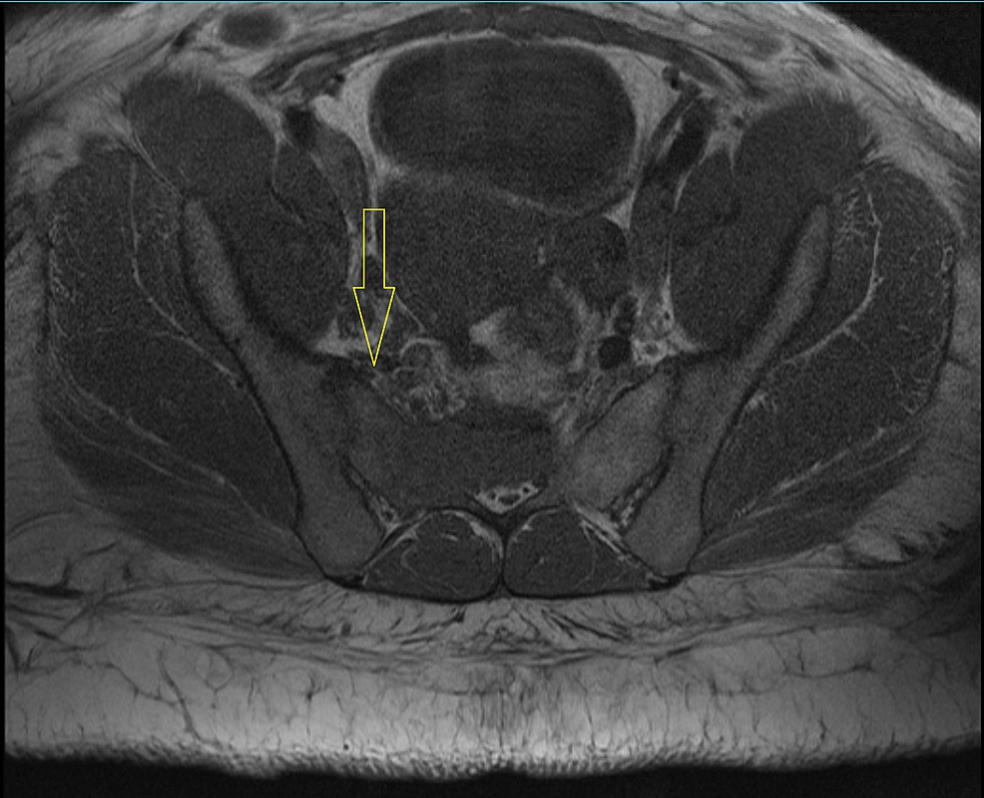
Magnetic resonance imaging of the lumbar spine showing a primarily centrally located sacral mass.

MRI of the lumbar spine also showed a sacral mass primarily centrally located extending between the right S1-2 and S2-3 sacral foramina (Figure 2).

Tumor markers carcinoembryonic antigen (CEA), carbohydrate antigen (CA)-19-9, and alfa fetoprotein (AFP) were within the normal range. Pheochromocytoma was ruled out. Before the workup could be completed, the patient left against medical advice and did not follow up for further management.

Five months later, the patient presented to the emergency department with severe low back pain radiating to the left flank for two days. There was decreased power (3/5) in the left lower extremity. A repeat MRI of the lumbosacral spine redemonstrated the sacrum mass which now had a more expansile appearance with an extraosseous extension in S2-4 vertebrae.

**Figure 3:**
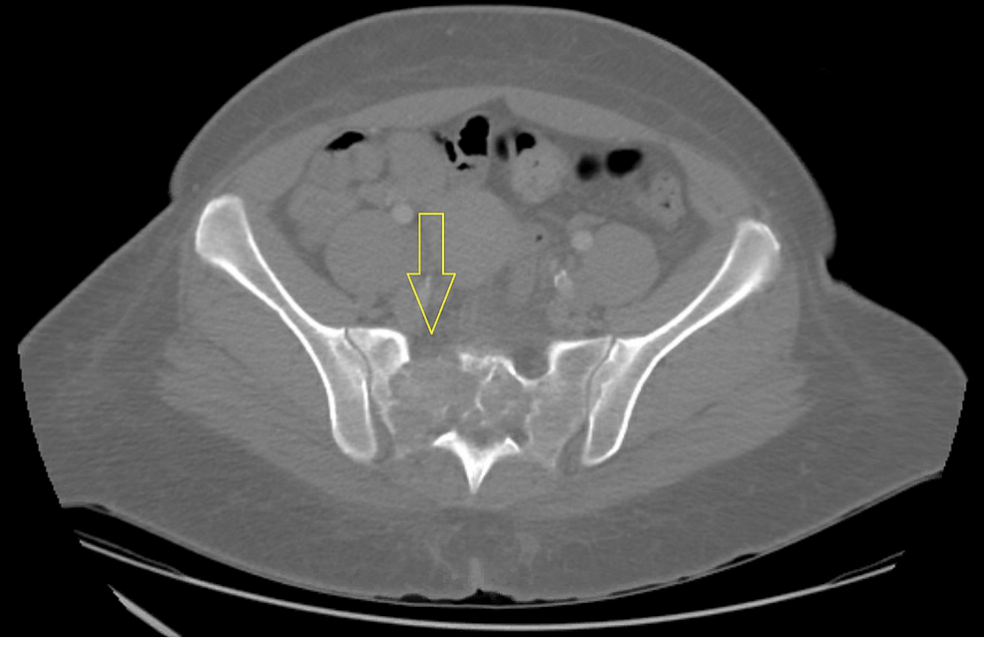
Computed tomography of the pelvis showing interval enlargement of the right sacral lytic mass crossing the midline and eroding anterior and posterior cortex of the sacrum.

The main differential diagnoses considered were chordoma, giant cell tumor, and metastatic disease. Multiple myeloma was also considered, but clinical and laboratory findings were not suggestive of multiple myeloma. A sacral bone biopsy was performed. While waiting for the results of the biopsy, a CT of the chest, abdomen, and pelvis with intravenous contrast was done which showed progression of osteolytic and destructive lesions in the sacrum associated with large soft-tissue components and stable bilateral inguinal adenopathy (Figure 3).

**Figure 4:**
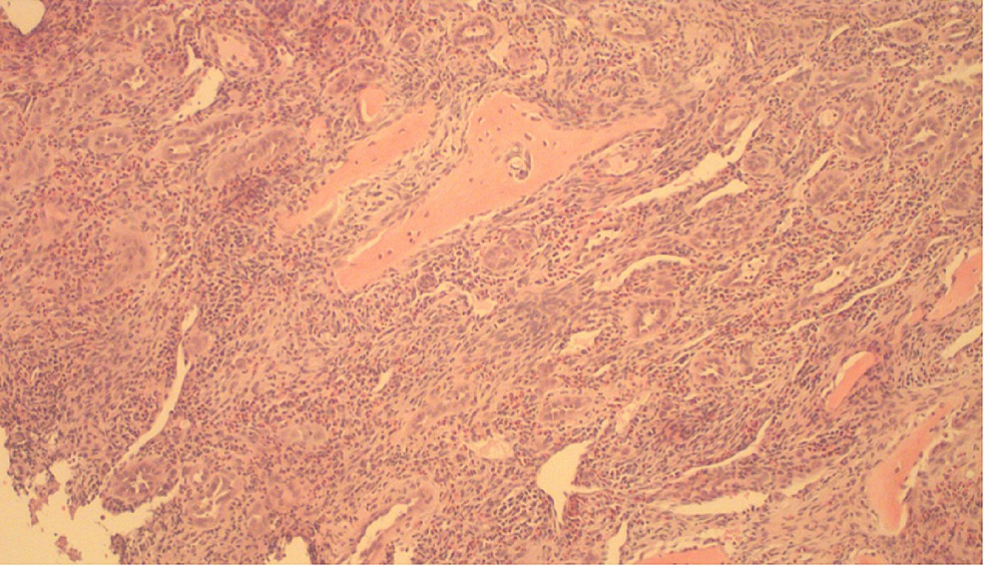
Core biopsy of the bone showing extensive vascular proliferation (hematoxylin and eosin, 10×).

**Figure 5:**
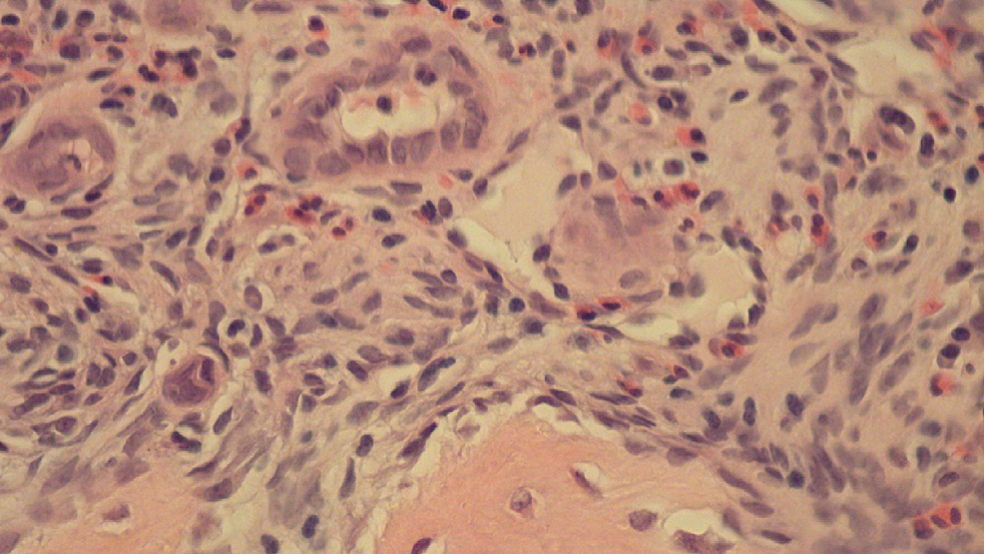
Bone showing vascular proliferation with increased eosinophils (hematoxylin and eosin, 50×).

**Figure 6:**
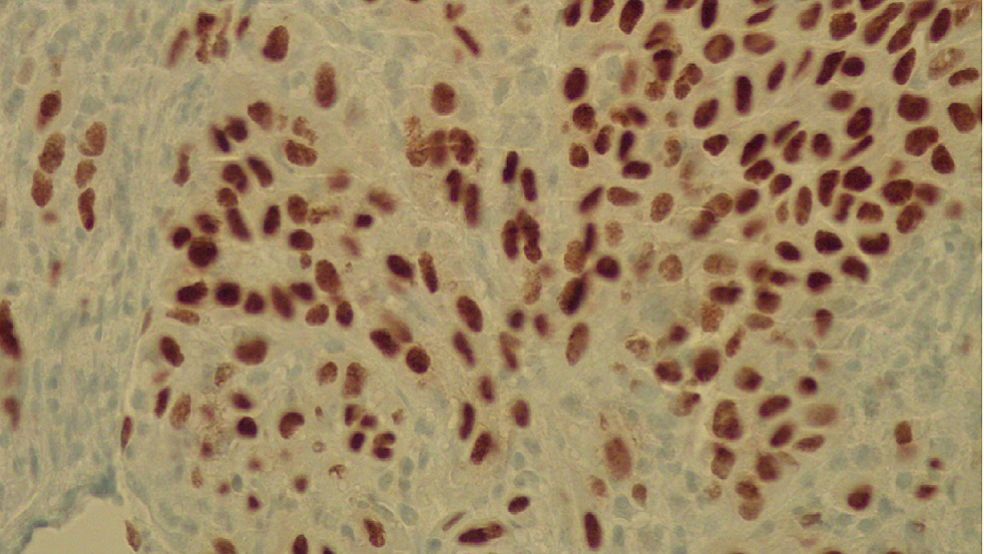
ERG immunohistochemical stain confirming endothelial cells (immunohistochemistry, 50×).

A preliminary pathology report showed concern for metastatic adenocarcinoma. Slides were sent to another referral center for a second opinion. Immunohistochemical stains were negative for AE1/AE3, cytokeratin (CK)7, CK20, paired-box gene 8 (PAX8), GATA binding protein 3 (GATA3), Mammaglobin, gross cystic disease fluid protein 15 (GCDFP-15), S100, thyroid transcription factor-1 (TTF-1), Napsin A, estrogen receptor (ER), progesterone receptor (PR), and human epidermal growth factor receptor 2 (Her2) but positive for CD31 and ETS-related gene (ERG), confirming the endothelial differentiation. Ki-67 stain showed a low proliferative rate of 5% (Figures 4-6).

Overall, the findings were consistent with an EH. The patient was referred to a higher center for a sacrectomy.

## Discussion

When a patient presents with a vertebral mass, metastatic cancer versus primary tumor is the main concern. Metastatic tumors are the most common tumors of the spine (97%) [9]. The most common primary malignancies metastasizing to the spine include the breast (21%), lung (19%), prostate (7.5%), renal (5%), gastrointestinal (4.5%), and thyroid (2.5%) [10]. However, in up to 30% of patients who present with bone metastases, the site of the primary neoplasm cannot be identified at the time of diagnosis despite an extensive workup [11]. Primary tumors of the spine are rare. Because most are asymptomatic, their real incidence remains unknown [9]. Primary sacral malignant tumors consist of sacral chordoma, chondrosarcoma, Ewing sarcoma/primitive neuroectodermal tumors (PNET), osteosarcoma, and multiple myeloma/plasmacytoma. Common benign tumors include giant cell tumor (GCT), aneurysmal bone cyst, osteoid osteoma, osteoblastoma, and cavernous hemangioma of the sacrum [12].

In our case, apart from a metastatic tumor, chordoma and GCT, being the two most common primary sacral tumors, were our top differential diagnoses. In chordoma, an X-ray shows osteolytic lesions of the sacrum with mass and calcifications in soft tissue. On CT scans, bone destruction with an associated lobulated midline soft-tissue mass is typically seen [13]. MRI shows high signal intensity heterogenous sacral mass with crisscrossing septa, well-encapsulated, and gluteal muscle infiltration [14]. In the case of GCTs, plain radiographs are the mainstay of diagnosis. Plain X-rays show cortical thinning, expansile remodeling, and cortical breakthrough with frequent trabeculation; a pathological fracture and periosteal reaction might be seen. On CT scans, GCTs present as eccentrically located solitary lucent bone lesions [15]. An MRI is usually nonspecific. Hemorrhagic area, bone marrow edema, soft-tissue extension, and solid tissue components may be seen [16].

EH is a mesenchymal tumor of vascular origin. The presence of osseous EH involving the spine is less common. As in our case, the EH of the spine may have locally aggressive features with progressive expansile nature. It may present with pain and/or neurological impairment due to the destruction of the bone causing instability or pathologic fractures [2]. Plain radiographs show expansile radiolucent, lytic, or cystic-appearing lesions, narrow transition zone, and endosteal scalloping. Similar to plain radiographs, CT scans reveal expansile lytic or cystic lesions with soft-tissue internals that are usually slightly lower than muscle density and without calcified or osteoid matrix. MRI usually shows lobulated lesions with intralesional flow voids; otherwise, features of EH are nonspecific with low-to-intermediate signal intensity on T1WI, heterogeneous, and intermediate-to-high signal intensity on T2WI [3]. Soft-tissue extension beyond the osseous lesions, as in our case, has also been reported. Bone scan uptake may be increased or normal [2].

Histologically, the proliferation of blood vessels with epithelial endothelial cells is observed. Endothelial cells are so dense that vascular spaces are sometimes difficult to assess and aggregates can look like granulomas. A collection of lymphocytes and numerous eosinophils accompany vascular proliferation [17]. Immunohistochemical markers may be positive for CD31, CD34, FOSB, ERG, FLI1, factor VIII, AE1/AE3, and smooth muscle actin (SMA). FOS rearrangement can be seen in one-third of EH cases. A typical variant of EH can show a FOS-LAMIN A/C (LMNA) fusion transcript, and, in the case of an atypical variant, ZFP36-FOSB fusions may be seen [2].

Various treatment options such as en bloc resection, curettage, pre/postoperative embolization, radiation, and microwave ablation have been used either alone or in combination with success. Local recurrence was reported in 11% of patients in a series of 36 patients with EH of bone treated with curettage, local resection, and radiotherapy [2]. In such cases, oral propranolol can be tried as there are some case reports where it has been successful in preventing progression and even decreasing the size of the tumor [18-20]. Imaging surveillance is recommended to assess recurrence [2].

## Conclusions

Whenever there is a radiological finding of a sacral mass, either with or without neurological involvement and/or low back pain, EH, although very rare, should be in our differential diagnoses. EH initially can be an incidental finding. However, patients should undergo a prompt evaluation as EH may become symptomatic gradually with pain and weakness from neurological impairment due to extraosseous extension of the tumor.
